# Huangqin Qingre Chubi Capsule inhibits rheumatoid arthritis by regulating intestinal flora and improving intestinal barrier

**DOI:** 10.3389/fphar.2024.1422245

**Published:** 2024-06-26

**Authors:** Yanhui Peng, Yurong Huang, Hui Li, Chen Li, Yajie Wu, Zhe-Sheng Chen, Xiao Wang, Faxue Liao, Chenggui Miao

**Affiliations:** ^1^ Department of Pharmacology, School of Integrated Chinese and Western Medicine, Anhui University of Chinese Medicine, Hefei, China; ^2^ Department of Pharmaceutical Sciences, College of Pharmacy and Health Sciences, St. John’s University, New York, United States; ^3^ Department of Clinical Nursing, School of Nursing, Anhui University of Chinese Medicine, Hefei, China; ^4^ Department of Orthopaedics, The First Affiliated Hospital, Anhui Public Health Clinical Center, Anhui Medical University, Hefei, China; ^5^ Institute of Prevention and Treatment of Rheumatoid Arthritis, Anhui University of Chinese Medicine, Hefei, Anhui, China; ^6^ School of Chinese Medicine, Li Ka Shing Faculty of Medicine, The University of Hong Kong, Hefei, China

**Keywords:** rheumatoid arthritis, lipolyaccharide, Huangqin Qingre Chubi Capsule, intestinal flora, intestinal barrier

## Abstract

**Background:**

Changes in intestinal flora and intestinal barrier in patients with preclinical and diagnosed rheumatoid arthritis (RA) suggest that intestinal flora and intestinal barrier play an important role in the induction and persistence of RA. Huangqin Qingre Chubi Capsule (HQC) is a clinically effective herbal formula for the treatment of RA, but its therapeutic mechanism has not been fully clarified.

**Materials and methods:**

In this study, real-time qPCR (RT-qPCR), 16SrRNA sequencing, Western blot (WB), immunofluorescence and other methods were used to investigate whether HQC inhibited RA.

**Results:**

Based on research in collages-induced arthritis (CIA) model in mice, human colon cancer cell line (Caco-2), and fibroblast-like synoviocytes (FLS) from RA patients, we found that intestinal flora was disturbed in CIA model group, intestinal barrier was damaged, and lipolyaccharide (LPS) level was increased, and HQC could regulate intestinal flora and intestinal barrier and reduce LPS translocation into blood. Antibiotic depletion weakened the anti-RA effect of HQC, and HQC fecal microbiota transplantation alleviated RA pathology. In addition, LPS increased the expression of RA pathologic factors MMP3, Fibronectin and inflammatory factors IL-6, TNF-α, IL-1β and IL-8, indicating that elevated peripheral blood level of LPS was related to RA pathology.

**Conclusion:**

The dysregulation of intestinal flora and the disruption of intestinal barrier are significant factors in the development of RA. HQC improves RA by regulating intestinal flora, intestinal barrier and inhibiting LPS translocation into blood. The study unveiles RA’s new pathogenesis and laid a scientific groundwork for advancing HQC therapy for RA.

## 1 Introduction

Rheumatoid arthritis (RA) is a systemic inflammatory disease that affects joints (Smolen et al., 2016). The occurrence of RA is related to genetic and environmental factors (Espinoza and García-Valladares, 2013; Taneja, 2015). Among environmental factors, intestinal flora has become an important environmental factor for the onset of RA (Bernard, 2014). Before the onset of RA, RA autoantibodies exist in the blood, indicating that the occurrence of RA may occur earlier than different parts of the joint, such as the gastrointestinal tract (Deane and Holers, 2021).

Mammals’ guts are home to a diverse population of bacteria known as the gut microbiota. The intestinal flora of mammals consists mainly of bacteria. The intestinal flora are crucial in preserving the balance of the immune system, dysfunction of intestinal flora can lead to immune inflammation and changes in intestinal barrier function, resulting in the emergence of illnesses like RA (Zaiss et al., 2021). The intestinal barrier can prevent harmful substances from entering the human body through the intestinal mucosa. The intestinal barrier is primarily maintained through a balance between the adhesive and tight junctions of epithelial cells (Groschwitz and Hogan, 2009; Capaldo et al., 2017). Impairment of the intestinal barrier may be another important mechanism for converting autoimmunity into inflammation.

Lipopolysaccharide (LPS) is made up of polysaccharides and lipids and forms an integral part of the cell wall in Gram-negative bacteria, it can act as an inducer of inflammation, thereby triggering the release of inflammatory cytokines (Xie et al., 2024). LPS can increase renal tubular injury and cell apoptosis, induce mitochondrial function and dynamic disorders (Jian et al., 2023). Serum LPS levels are low in healthy people. When intestinal flora is disturbed, especially when Gram-negative bacteria overmultiply to produce large amounts of LPS, the serum LPS level will significantly increase. The impaired intestinal barrier further increases LPS translocation into the blood (Chen et al., 2021). The role of intestinal flora disturbance and intestinal barrier injury in increasing LPS entry into peripheral blood, as well as their relationship with the onset of RA. This is a question worthy of in-depth exploration.

Fibroblast-like synoviocytes (FLS) preserve synovial fluid and extracellular matrix balance in healthy joints by producing joint and synovial fluid components (Jay et al., 2001). Excessive proliferation of FLS plays a central role in the pathogenesis of RA. Activated FLS secrete pro-inflammatory factors, triggering the development of excessive synovial growth and inflammation (Karami et al., 2020).

The human colon cancer cell line (Caco-2) is often used as a cell model for the absorption of drugs and other compounds in the human intestine. Caco-2 cells were initially isolated from human colorectal adenocarcinoma and spontaneously differentiated into intestinal epithelial cells during culture, similar to those of the small intestine (Hilgers et al., 1990). When the cells grow to a fusion state, the Caco-2 cell monolayer forms cell polarity and robust connections, with multiple active transport mechanisms active in the human small intestine walls. The apparent permeability coefficient of the measured reference compound through the single-layer membrane of Caco-2 cells has a good correlation with *in vivo* absorption (Artursson and Karlsson, 1991). Because of these favorable properties, the Caco-2 cell monolayer experiments are now the benchmark *in vitro* method for assessing the intestinal permeability and movement of potential drugs and primary compounds.

Huangqin Qingre Chubi Capsule (HQC) was developed by the First Affiliated Hospital of Anhui University of Chinese Medicine (patent No. ZL201110095718.X). HQC includes five Chinese medicinal herbs: Scutellaria baicalensis Georgi’s dry roots, *Gardenia jasminoides* J.Ellis’ dry mature seeds, *Coix lacryma-jobi* var. *stenocarpa’s* dry and mature seeds, Oliv.’s dry mature *Amygdalus persica* L. seeds, and the roots and rhizomes of *Clematis chinensis* Osbeck (ratio 10:9:30:5:10) (Zhou et al., 2023). HQC is a proprietary Chinese medicine for treating RA based on the theory of traditional Chinese medicine, which can alleviate RA by inhibiting FLS proliferation. HQC can significantly improve the feelings of RA patients and improve the symptoms of RA patients. However, the molecular mechanism has yet to be fully elucidated.

This study took intestinal flora and intestinal barrier as the entry point to explore whether HQC regulates the abundance of intestinal Gram-negative bacteria and intestinal barrier, thereby inhibiting LPS translocation into blood and improving RA. The study elucidates the mechanism of HQC improving RA by inhibiting intestinal flora and intestinal barrier, providing a scientific basis for the clinical promotion of HQC improving RA, and providing a new perspective for traditional Chinese medicine treatment of rheumatism.

## 2 Materials and methods

### 2.1 Preparation of CIA mice model

C57BL/6 mice, SPF grade, male, weight 22–24 g, adaptive feeding for 1 week. The emulsion was created using a high-speed stirrer by blending 2 mg/mL of chicken Type II collagen with 5 mg/mL of Freund complete adjuvant in the same quantity. The mice were injected with 0.1 mL emulsion at the tail root for the first immunization, and the second immunization was performed 21 days later, the same as the first modeling method (Murayama et al., 2015; Miyoshi and Liu, 2018). The animal welfare ethics review for this experiment was approved by Anhui University of Chinese Medicine’s Animal Ethics Committee (AHUCM-mouse-2023079, 7 July 2023).

### 2.2 Cell culture

Synovial tissue of knee joints of RA patients and non-RA patients was obtained from discarded synovial tissue after knee joint surgery. In the First Affiliated Hospital of Anhui Medical University (PJ-YX2021-026). The cultivation of human synovial FLS was conducted using the tissue block technique. Caco-2 cell line (iCELL-h032) was purchased from Cybiocon Biotechnology Co., LTD. (Shanghai, China), consists of 20% fetal bovine serum (Gibco, United States), high-sugar DMEM medium (Gibco, United States), and 1% penicillin-streptomycin solution (Beyotime, China). The human primary FLS used in this work were the third to sixth generation of cultured FLS (Wang X. et al., 2021).

### 2.3 Drug administration method and preparation of drug-containing serum

For the CIA mouse study, methotrexate (MTX Shanghai SINE Pharmaceutical Co., LTD., Lot No. 210502) served as a positive control medication. Based on the prescribed clinical dosage, the algorithm for exchanging body surface area between humans and mice, along with related references (Wang et al., 2022), we determined that MTX gavage dose was 0.75 mg/kg (twice a week), HQC daily dose was 0.36 g/kg, and gavage volume was 10 mL/kg. Three doses of HQC were set to 0.18 g/kg, 0.36 g/kg and 0.72 g/kg, respectively. The negative control group was given normal saline intragastric once a day. CIA mice were given LPS by intraperitoneal injection 48 h before sampling, with three doses of 2 mg/kg, 4 mg/kg and 8 mg/kg, respectively. The negative control group was intraperitoneally injected with normal saline (Shen et al., 2022).

The drug-containing serum was prepared by HQC (0.36 g/kg) and administrated by intragastric administration (10 mL/kg), the dose was divided into two times per day, and the control group was administrated with normal saline. Each group was given continuous administration for 5 days 45 min after the last administration, blood was taken from the inner canthus, centrifuged at 3,000 r/min for 10 min, filtered, and the complement was inactivated by a water bath at 56°C for 30 min. Mixed well in the same group, sealed, and stored at −80°C for later use. For the cell experiment, it was divided into normal group, Caco-2+LPS group, HQC low dose (5%) group, HQC medium dose (10%) group, HQC high dose (20%) group and negative control group (equal dose of normal serum).

### 2.4 Construction of pseudo-sterile mice and fecal microbiota transplantation (FMT)

To consume the intestinal flora of mice in the CIA model group and HQC treatment group, pseudo-sterile mice were prepared by continuous administration of neomycin sulfate (200 mg/kg), ampicillin (200 mg/kg), vancomycin (100 mg/kg) and metronidazole (200 mg/kg) once a day for 1 week (Dou et al., 2023). HQC treatment group (0.36 g/kg) mice and CIA mice as fecal donors. The recipient mice also selected the CIA mice that were successfully modeled. Fresh stool of donor mice was collected, and 600 mg of stool was immediately immersed in 6 mL of normal saline for 1 min. Dissolved fecal matter underwent centrifugation at 1,000 *g* (4°C) for 3 min, followed by the collection of the suspension. Then, donor fecal supernatant was immediately administrated into recipient mice (100 μL/mouse) for continuous gavage for 2 weeks (Zhao et al., 2021).

### 2.5 Scoring of arthritis

The following was a list of the precise requirements for the arthritis scores: Grade 0: lacking redness and swelling; Grade 1: redness and swelling of the small toe joints; Grade 2: redness and swelling of all the joints and toes; Grade 3: redness and swelling below the ankle joint. Grade 4: redness and swelling of all joints, including ankle joints. Each mouse’s arthritis index was determined by adding together the scores for each joint (Sun et al., 2023).

### 2.6 Histopathological examination

Mouse knee joints underwent fixation in 4% paraformaldehyde for a day and were then demineralized using a 10% ethylene diamine tetraacetic acid solution. Following the processes of dehydration using gradient alcohol, clarification, and embedding in paraffin, the samples were sequentially sectioned. Following the sections’ staining, dehydration, transparency, and sealing, microscopic examination revealed the histopathological alterations and intensity (Cheng et al., 2023) (CX41, OLYMPUS, Japan).

### 2.7 RT-qPCR

RNA extraction RNase was irreversibly denatured with Trizol reagent (Invitrogen, California, United States), and total RNA was precipitated with isopropyl alcohol. Incorporated 75% ethanol that has been pre-cooled, using a centrifuge, and subsequently mixed in DEPC water for dissolution. According to the instructions of the reverse transcription kit (biosharp, Hefei, China), the full reaction system of 13 μL was prepared, whirlpool centrifugation for 5 s, and cDNA was obtained by reverse transcription. The specific parameters are 25°C 10 min, 55°C 15 min, and 85°C 5 min. The RT-qPCR Kit (biosharp, Hefei, China) served as the tool for PCR amplification. Particular operational variables include: 95°C 10 min, 95°C 15 s, 60°C 60 s, 72°C 30 s, 40 cycles. Every gene underwent 3 times across 5 separate samples, followed by the computation of each group’s expression level using 2^−ΔΔCT^ (Wang et al., 2022). (The primers are enumerated in Table 1)

**TABLE 1:** Sequences of primers for RT-qPCR.

Sequences of primers for RT-qPCR (mouse)		
Gene	Forward primer	Reverse primer
ZO-1	TGC​TAA​TGC​CTC​GGA​AAG​AGA​TGA​C	GCT​GTG​GAG​ACT​GCG​TGG​AAT​G
Occludin	TGG​CTA​TGG​AGG​CGG​CTA​TGG	ACT​AAG​GAA​GCG​ATG​AAG​CAG​AAG​G
Claudin-1	GCT​GGG​TTT​CAT​CCT​GGC​TTC​TC	CCT​GAG​CGG​TCA​CGA​TGT​TGT​C
Zonulin	GCT​GTT​GTC​ACT​CTC​CTG​CTC​TG	CGG​CAG​CGA​TAG​CGA​ACC​AAG
MMP3	GAC​GAT​GAT​GAA​CGA​TGG​ACA​GAG	GCC​TTG​GCT​GAG​TGG​TAG​AGT​C
Fibronectin	CGA​AGT​CAG​TGT​CTA​TGC​TCT​CAA​G	GGT​CTC​TGT​AGC​GTC​CGT​CAC
β-actin	CTG​GCA​CCA​CAC​CTT​CTA​CAA​TGA​G	AGA​GGC​ATA​CAG​GGA​CAG​CAC​AG

Sequences of primers for RT-qPCR (human)		
Gene	Forward primer	Reverse primer
ZO-1	ACA​TTG​CCG​CCA​GCC​ATC​TC	ATC​TAT​CCA​CAC​CAT​CAG​CTT​CAG​G
Occludin	ACT​TCG​CCT​GTG​GAT​GAC​TTC​AG	TTC​TCT​TTG​ACC​TTC​CTG​CTC​TTC​C
Claudin-1	AGG​TAC​GAA​TTT​GGT​CAG​GCT​CTC	GGG​ACA​GGA​ACA​GCA​AAG​TAG​GG
Zonulin	GGC​ATT​ATG​AAG​GCA​GCA​CAG​TC	TCG​CCA​TAG​CAG​GTG​TCT​TCT​TG
β-actin	CCT​GGA​CTT​CGA​GCA​AGA​GAT​GG	CAG​GAA​GGA​AGG​CTG​GAA​GAG​TG

Primers were provided by Shanghai Sangon Biotechnology (Shanghai, China).

### 2.8 Western blot (WB)

Proteins were extracted from large and small intestine tissues, and Caco-2 cells were harvested from each group. Diluted the primary antibody with primary antibody diluent according to the antibody instructions, incubated PVDF membrane overnight at 4°C. After 1 h of incubation with the secondary antibody, rinsed three times. The protein bands were detected using an ECL kit (Biosharp, Hefei, China), analyzed using ImageJ software. Antibodies employed in this study, including Occludin (AB216327, 1:1,000), Claudin-1 (AB307692, 1:1,000) and β-actin (AB8226, 1:1,000), all acquired from Abcam Company (Waltham, Massachusetts, United States) (Wang et al., 2022).

### 2.9 Cell counting kit-8 (CCK-8)

Inserted FLS into a plate with 96 wells, allowed FLS to expand to 80% of the well’s lower region, and subsequently began incorporating drugs for evaluation. Following the CCK-8 kit’s guidelines (Beyotime, Shanghai, China), the impact of varying LPS dosages on FLS proliferation was identified, and the absorbance was measured at 450 nm using a microplate reader (Wang et al., 2022).

### 2.10 ELISA

ELISA (ColorfulGene, Wuhan, China) was used to measure the concentrations of interleukin (IL), TNF-α, rheumatoid factor (RF), C-reactive protein (CRP), and LPS. Mixed the standard substance with standard diluent in a suitable ratio and create a standard curve per the product manual. Set blank and sample holes, added sample, and added enzyme-labeled reagent except blank well. After incubation and washing, the developer solution was introduced, followed by a reaction in darkness and the addition of the stop solution. An enzyme labeled instrument was employed to ascertain the OD measurement. Every group recorded three samples, each undergoing three tests (Wang et al., 2022).

### 2.11 Immunofluorescence

FLS and Caco-2 were uniformly inoculated in 35 mm clear glass cell culture dishes (Corning, NY, United States) until the cells were completely attached to the wall. The cells underwent fixation using 4% paraformaldehyde followed by a rinse in PBS. At ambient temperature, the FLS underwent infiltration with 0.1% Triton X-100, then were sealed using 1% BSA, followed by treatment with primary antibodies and incubation with suitable secondary antibodies. Nuclei samples underwent staining using DAPI in darkness. After washing with PBS, sealed with fluorescence quencher (Wang et al., 2022). Images were captured using a fluorescence microscope. ZO-1 antibody (ab221547, 1:100), MMP3 antibody (ab52915, 1:250) and Fibronectin antibody (ab268020, 1:200) all were acquired from Abcam Company (Waltham, Massachusetts, United States). Zonulin antibody (DF6467, 1:100) was purchased from Affinity Biosciences Company (Affinity Biosciences, OH, United States).

### 2.12 DNA extraction and 16SrRNA sequencing

The mice received anesthesia through an intraperitoneal administration of 50 mg/kg of 0.3% pentobarbital sodium. Blood was collected from the inner canthus of mice in each group. Fresh samples of small and large intestine contents (0.3 g for each sample) were collected and immediately stored at −80°C. Sample extraction of DNA, detection of DNA concentration and purity. PCR amplification of bacterial 16S rRNA gene used DNA extraction as a template, and amplified the V3-V4 variable region of the 16SrRNA gene for bacterial diversity analysis. PCR amplification product was detected and purified. After purification, amplification product was used as a second round PCR template for second round PCR amplification and further purification. The purified second round product was quantified by Qubit, and adjustments were made to the concentration for sequencing purposes. Sequencing was performed using the Illumina NovaSeq 6,000 sequencing platform and 250 bp double-ended reads were generated. Sequencing was performed by Shanghai Ouyi Biotechnology Co., LTD. (Shanghai, China) (Nossa et al., 2010).

### 2.13 Statistical analysis

The data analysis was conducted using the SPSS 26.0 statistical program. The mean values across various groups were compared using a one-way analysis of variance, followed by a *post hoc* LSD test for additional pairwise comparisons. The *t*-test used a comparative analysis between the two groups. The data were expressed as mean ± SD, and *p* < 0.05 was considered significant.

## 3 Results

### 3.1 HQC alleviated the pathology of CIA mice

**FIGURE 1:**
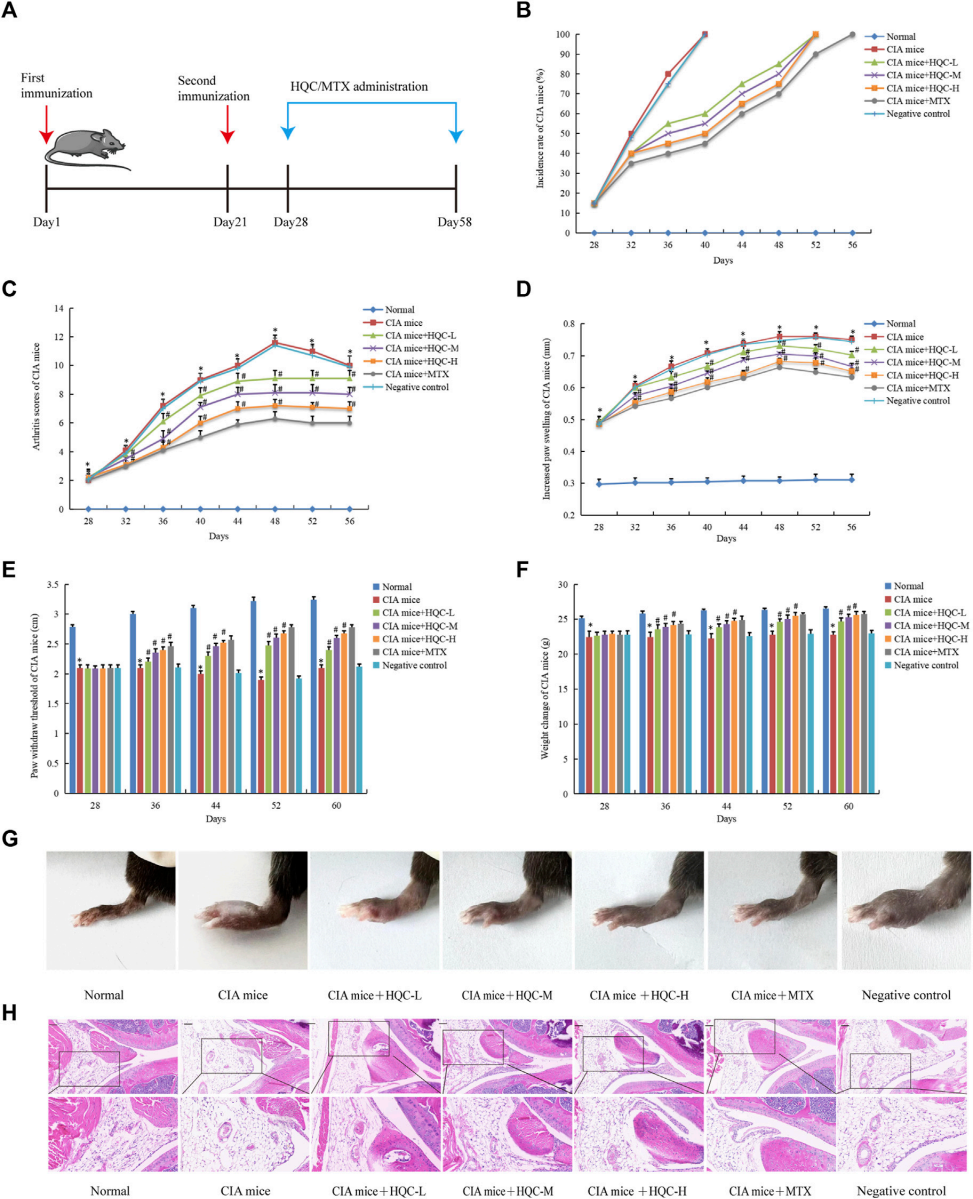
HQC alleviated the pathology of CIA mice **(A)**, The chart of animal treatments; **(B)**, Incidence; **(C)**, Arthritis score; **(D)** Foot swelling; **(E)**, Foot withdrawal thresholds; **(F)**, Body weight; **(G)**, Foot swelling picture; **(H)**, HE staining of ankle joint tissue. *p < 0.05 vs. Normal, #*p* < 0.05 vs. CIA mice; for **(B–F)**, *n* = 10; for **(G and H)**, *n* = 3.

CIA mice were administered for 30 days (Figure 1A). In order to evaluate the efficacy of HQC on CIA mice, we used the incidence rate, joint score, degree of hind foot swelling, threshold of foot withdrawal and body weight as evaluation indicators. Compared with the model group, the HQC group had a significant delay in reaching 100% incidence (Figure 1B). Arthritis score (Figure 1C) and foot swelling (Figure 1D) in the model group were significantly higher than in the normal group, reaching a peak at 48 days. From the eighth day of HQC administration, there was a notable decrease in the aforementioned indices within the HQC and MTX groups. Ankle pain sensitivity in CIA mice was assessed using foot withdrawal thresholds (Figure 1E), and body weight changes were measured to assess health status in each group (Figure 1F). Mice in the HQC and MTX groups exhibited a notably greater threshold for foot withdrawal and a higher body mass index than those in the model group. Foot swelling pictures of mice in each group on the 60th day were shown (Figure 1G).

In the normal group, H&E staining revealed clear synovial tissue in the knee joints, devoid of hyperplasia, and the joint’s exterior was sleeked, with both the articular cartilage and the subchondral bone framework being intact. In the CIA model group, the synovial tissue exhibited hyperplasia, the articular surface showed irregularity, pannus formation, and both the cartilage and subchondral bone suffered significant damage. Compared with model group, synovial hyperplasia, inflammatory cell infiltration and pannus formation of CIA mice in HQC and MTX groups were significantly reduced (Figure 1H).

### 3.2 HQC regulated the intestinal flora in the large intestine of CIA mice

**FIGURE 2:**
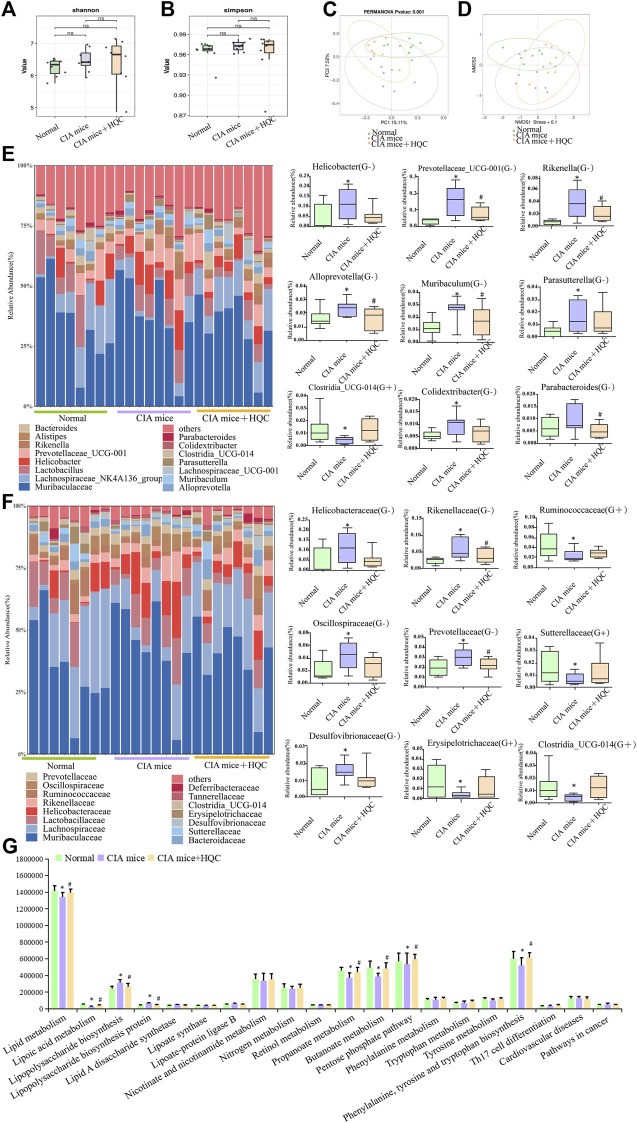
HQC regulated the intestinal flora in the large intestine of CIA mice **(A)**, Shannon analysis of the intestinal flora in the large intestine; **(B)**, Simpson analysis of the intestinal flora in the large intestine; **(C)**, PCoA analysis of the intestinal flora in the large intestine; **(D)**, NMDS analysis of the intestinal flora in the large intestine; **(E)**, Relative abundance of intestinal flora at the genus level and the relative abundance of nine significantly altered bacterial genera; **(F)**, Relative abundance of intestinal flora at the family level and the relative abundance of nine significantly altered bacterial families; **(G)**, Functional prediction chart. *p < 0.05 vs. Normal, #*p* < 0.05 vs. CIA mice, *n* = 8.

In the 16SrRNA sequencing results of large intestine contents samples, the Shannon index and the Simpson index demonstrated no significant difference in α diversity between the three groups (Figures 2A,B). The PCoA and NMDS analyses demonstrated significant differences in β diversity between the three groups (Figures 2C,D). At the genus level, *Muribaculaceae*, *Lachnospiraceae_NK4A136_group, Helicobacter, Lactobacillus, Alistipes*, *Prevotellaceae_UCG-001, Bacteroides, Muribaculum, Rikenella, Alloprevotella, Lachnospiraceae_UCG-001, Clostridia_UCG-014, Parasutterella, Parabacteroides* and *Colidextribacter* were the main genus of bacteria in the three groups of samples of large intestine contents*.* Compared with the normal group, the relative abundance of Gram-negative bacteria *Helicobacter, Prevotellaceae_UCG-001, Rikenella, Alloprevotella, Muribaculum, Parasutterella, Colidextribacter* and *Parabacteroides* were increased in the CIA model group, the relative abundance of Gram-positive bacteria *Clostridia_UCG-014* was decreased in the CIA model group, HQC treatment can partially restore the changes in the above indicators (Figure 2E). At the family level, *Muribaculaceae*, *Lachnospiraceae*, *Helicobacteraceae*, *Prevotellaceae*, *Rikenellaceae*, *Ruminococcaceae*, *Lactobacillaceae*, *Oscillospiraceae*, *Bacteroidaceae*, *Desulfovibrionaceae*, *Clostridia_UCG-014*, *Sutterellaceae*, *Erysipelotrichaceae*, *Tannerellaceae* and *Deferribacteraceae* were the main family of bacteria in the three groups of samples of large intestine contents. Compared with the normal group, the relative abundance of Gram-negative bacteria *Helicobacteraceae*, *Rikenellaceae*, *Oscillospiraceae*, *Prevotellaceae* and *Desulfovibrionaceae* were increased in the CIA model group, the relative abundance of Gram-positive bacteria *Ruminococcaceae*, *Sutterellaceae*, *Erysipelotrichaceae and Clostridia_UCG-014* were decreased in the CIA model group, HQC treatment partially restored the changes in the above indicators (Figure 2F). The PICRUST software predicted the function of the large intestine contents and found that the LPS generation path in mice was significantly attenuated after HQC intervention (Figure 2G).

### 3.3 HQC regulated the intestinal flora in the small intestine of CIA mice

**FIGURE 3:**
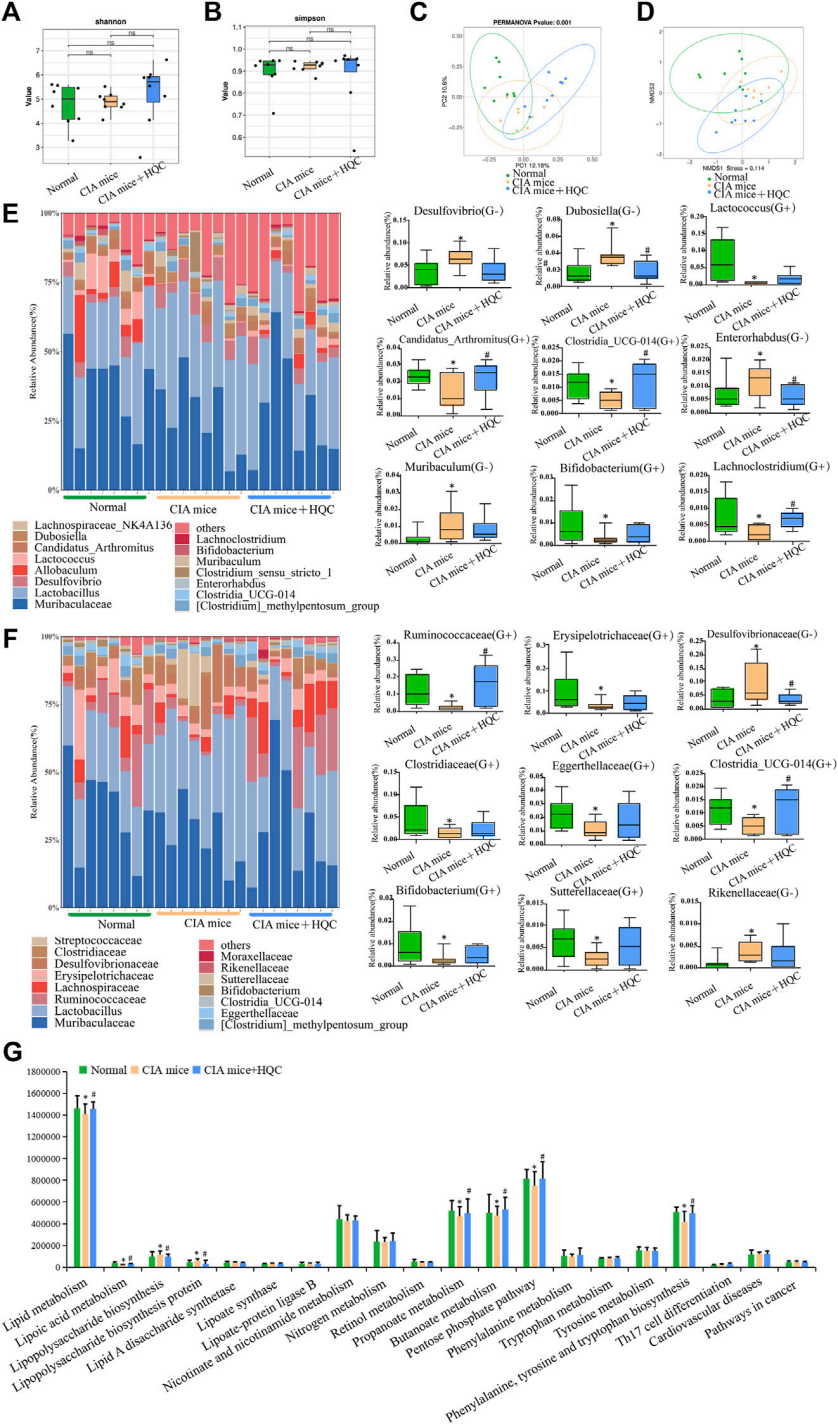
HQC regulated the intestinal flora in the small intestine of CIA mice **(A)**, Shannon analysis of the intestinal flora in the small intestine; **(B)**, Simpson analysis of the intestinal flora in the small intestine; **(C)**, PCoA analysis of the intestinal flora in the small intestine; **(D)**, NMDS analysis of the intestinal flora in the small intestine; **(E)**, Relative abundance of intestinal flora at the genus level and the relative abundance of nine significantly altered bacterial genera; **(F)**, Relative abundance of intestinal flora at the family level and the relative abundance of nine significantly altered bacterial families; **(G)**, Functional prediction chart. *p < 0.05 vs. Normal, #*p* < 0.05 vs. CIA mice, *n* = 8.

In the 16S RNA sequencing results of small intestine contents samples, the Shannon index and the Simpson index also demonstrated no significant difference in α diversity between the three groups (Figures 3A,B). The PCoA and NMDS analyses also demonstrated significant differences in β diversity between the three groups (Figures 3C,D). At the genus level, *Muribaculaceae*, *Lactobacillus*, *Desulfovibrio*, *Dubosiella*, *Lachnospiraceae_NK4A136_group*, *Allobaculum*, *Lactococcus*, *Candidatus_Arthromitus [Clostridium]_methylpentosum_group*, *Clostridia_UCG-014*, *Enterorhabdus*, *Clostridium_sensu_stricto_1*, *Muribaculum*, *Bifidobacterium and Lachnoclostridium are the main genus of bacteria in the three groups of samples of large intestine contents.* Compared with the control group, the relative abundance of Gram-negative bacteria *Desulfovibrio*, *Dubosiella*, *Enterorhabdus* and *Muribaculum* were increased in the CIA model group, the relative abundance of Gram-positive bacteria *Lactococcus*, *Candidatus_Arthromitus*, *Clostridia_UCG-014*, *Bifidobacterium* and *Lachnoclostridium* were decreased in the CIA model group, HQC treatment can partially restore the changes in the above indicators (Figure 3E). At the family level, *Muribaculaceae*, *Lactobacillaceae*, *Ruminococcaceae*, *Lachnospiraceae*, *Erysipelotrichaceae*, *Desulfovibrionaceae*, *Clostridiaceae*, *Streptococcaceae [Clostridium]_methylpentosum_group*, *Eggerthellaceae*, *Clostridia_UCG-014*, *Bifidobacteriaceae*, *Sutterellaceae*, *Moraxellaceae* and *Rikenellaceae* were the main family of bacteria in the three groups of samples of large intestine contents*.* Compared with the control group, the relative abundance of Gram-negative bacteria *Desulfovibrionaceae* and *Rikenellaceae* were increased in the CIA model group, the relative abundance of Gram-positive bacteria *Ruminococcaceae*, *Erysipelotrichaceae*, *Clostridiaceae*, *Eggerthellaceae*, *Clostridia_UCG-014*, *Bifidobacteriaceae* and *Sutterellaceae* were decreased in the CIA model group, HQC treatment can partially restore the changes in the above indicators (Figure 3F). The PICRUST software predicted the function of the small intestine contents and found that the LPS generation path in mice was significantly attenuated after HQC intervention (Figure 3G).

### 3.4 HQC treatment restored intestinal barrier damage in the large intestine of CIA mice

**FIGURE 4:**
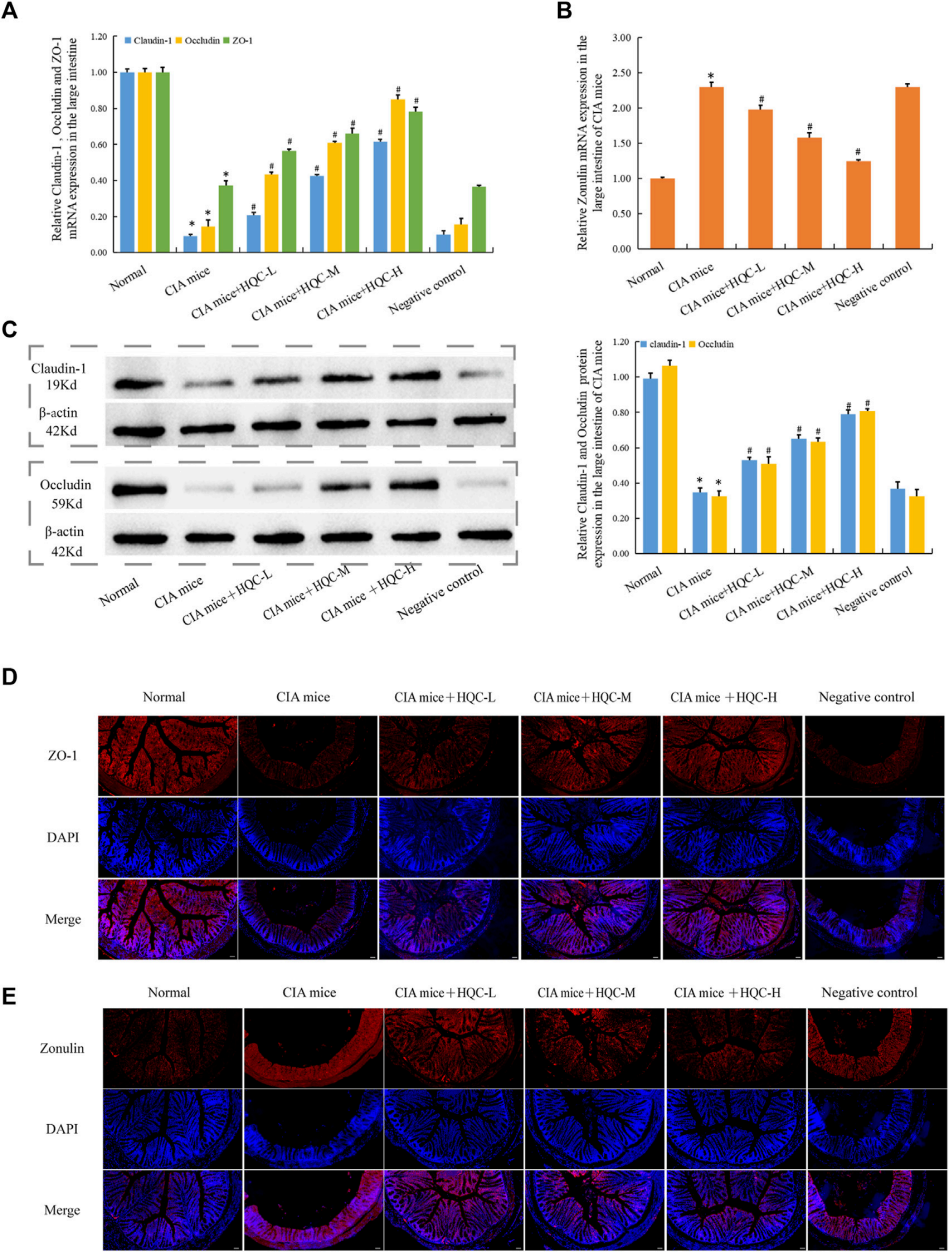
HQC treatment restored intestinal barrier damage in the large intestine in CIA mice **(A)**, The mRNA expression of ZO-1, Occludin, and Claudin-1 in large intestine tissue; **(B)**, The mRNA expression of Zonulin in large intestine tissue; **(C)** The protein expression of Occludin, and Claudin-1 in large intestine tissue; **(D)**, Immunofluorescence detection of ZO-1 expression in large intestine tissue (scale bar = 50 μm); **(E)**, Immunofluorescence detection of Zonulin expression in large intestine tissue (scale bar = 50 μm); *p < 0.05 vs. Normal, #*p* < 0.05 vs. CIA mice; for **(A–C)**, *n* = 5; for **(D and E)**, *n* = 3.

In the large intestine, compared with the normal group, the mRNA expression of ZO-1, Occludin and Claudin-1 in the CIA model group decreased (Figure 4A), the mRNA expression of Zonulin increased (Figure 4B), and HQC administration reversed the above indicators. Compared with the normal group, the protein expression of Occludin and Claudin-1 in the CIA model group decreased (Figure 4C), and HQC administration upregulated the expression of these two proteins in the large intestine of CIA mice. Immunofluorescence detection results showed that the expression of ZO-1 was decreased (Figure 4D) and Zonulin (Figure 4E) was increased in the CIA model group, and the expression of these two proteins was reversed by HQC administration. Compared with the normal group, the intestinal barrier was damaged in the CIA model group, and HQC treatment could alleviate the intestinal barrier damage in the large intestine of CIA mice.

### 3.5 HQC treatment restored intestinal barrier damage in the small intestine of CIA mice

**FIGURE 5:**
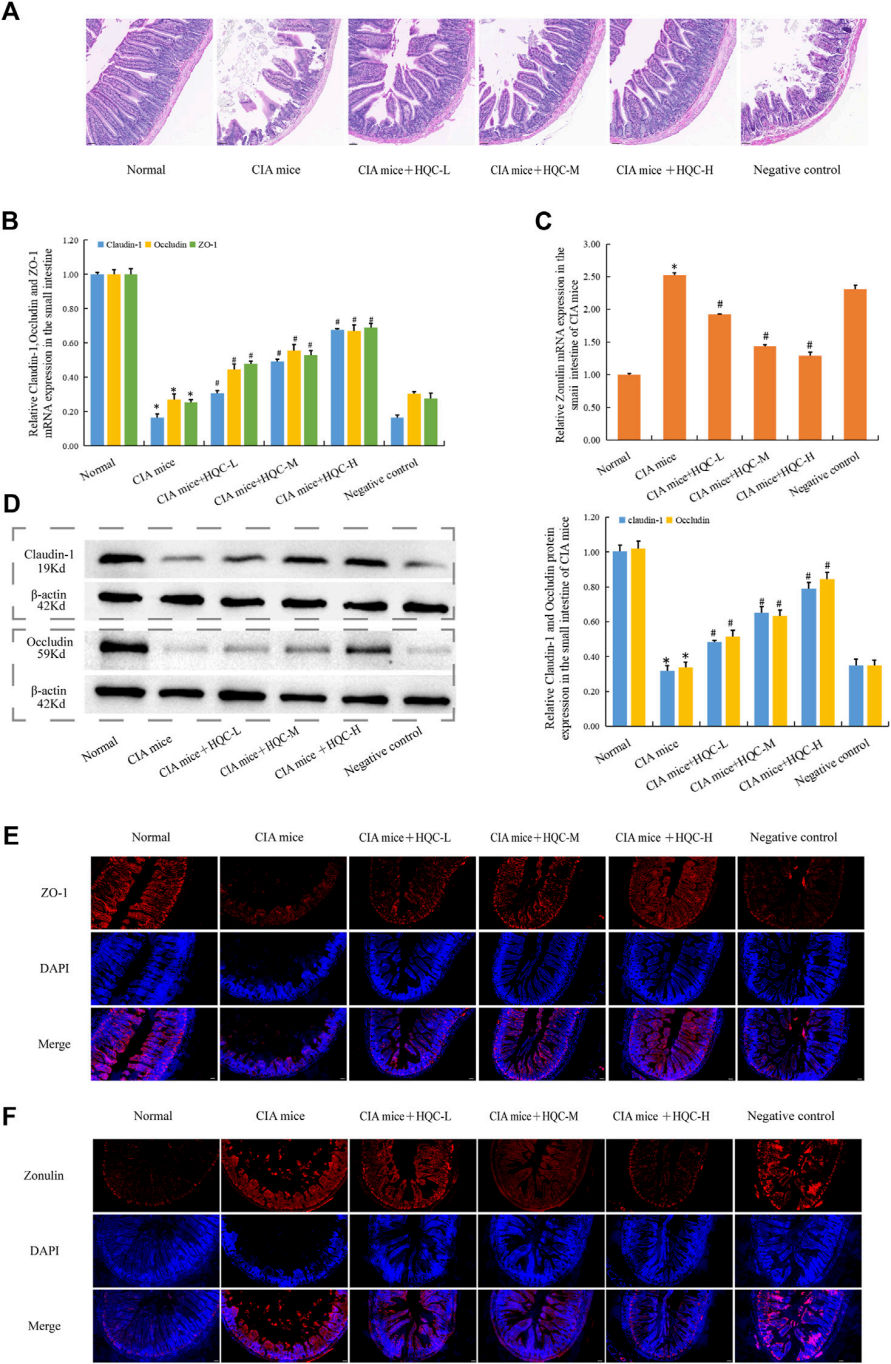
HQC treatment restored intestinal barrier damage in the small intestine of CIA mice **(A)**, HE staining of small intestine tissue; **(B)**, The mRNA expression of ZO-1, Occludin, and Claudin-1 in small intestine tissue; **(C)**, The mRNA expression of Zonulin in small intestine tissue; **(D)**, The protein expression of Occludin, and Claudin-1 in small intestine tissue; **(E)**, Immunofluorescence detection of ZO-1 expression in small intestine tissue (scale bar = 50 μm); **(F)**, Immunofluorescence detection of Zonulin expression in small intestine tissue (scale bar = 50 μm). *p < 0.05 vs. Norma, #*p* < 0.05 vs. CIA mice; for A, *n* = 3; for **(B–D)**, *n* = 5; for **(E and F)**, *n* = 3.

H&E staining of the small intestine showed that compared with the normal group, the intestinal villi in the model group were arranged sparsely and disordered with varying lengths, while the intestinal villi in the HQC treatment group were arranged more neatly with the same lengths (Figure 5A). Compared with the normal group, the mRNA expression of ZO-1, Occludin and Claudin-1 in CIA model group decreased (Figure 5B), the mRNA expression of Zonulin increased (Figure 5C), and HQC administration reversed the above indicators. Compared with the normal group, the protein expression of Occludin and Claudin-1 in the CIA model group decreased (Figure 5D), and HQC upregulated the expression of these two proteins. Immunofluorescence detection results showed that the expression of ZO-1 was decreased (Figure 5E) and Zonulin was increased (Figure 5F) in the CIA model group, and HQC treatment reversed the above indexes. Compared with the normal group, the intestinal barrier was damaged in the CIA model group, and HQC treatment could alleviate the intestinal barrier damage in the small intestine of CIA mice.

### 3.6 HQC treatment restored intestinal barrier damage in Caco-2 cells

**FIGURE 6:**
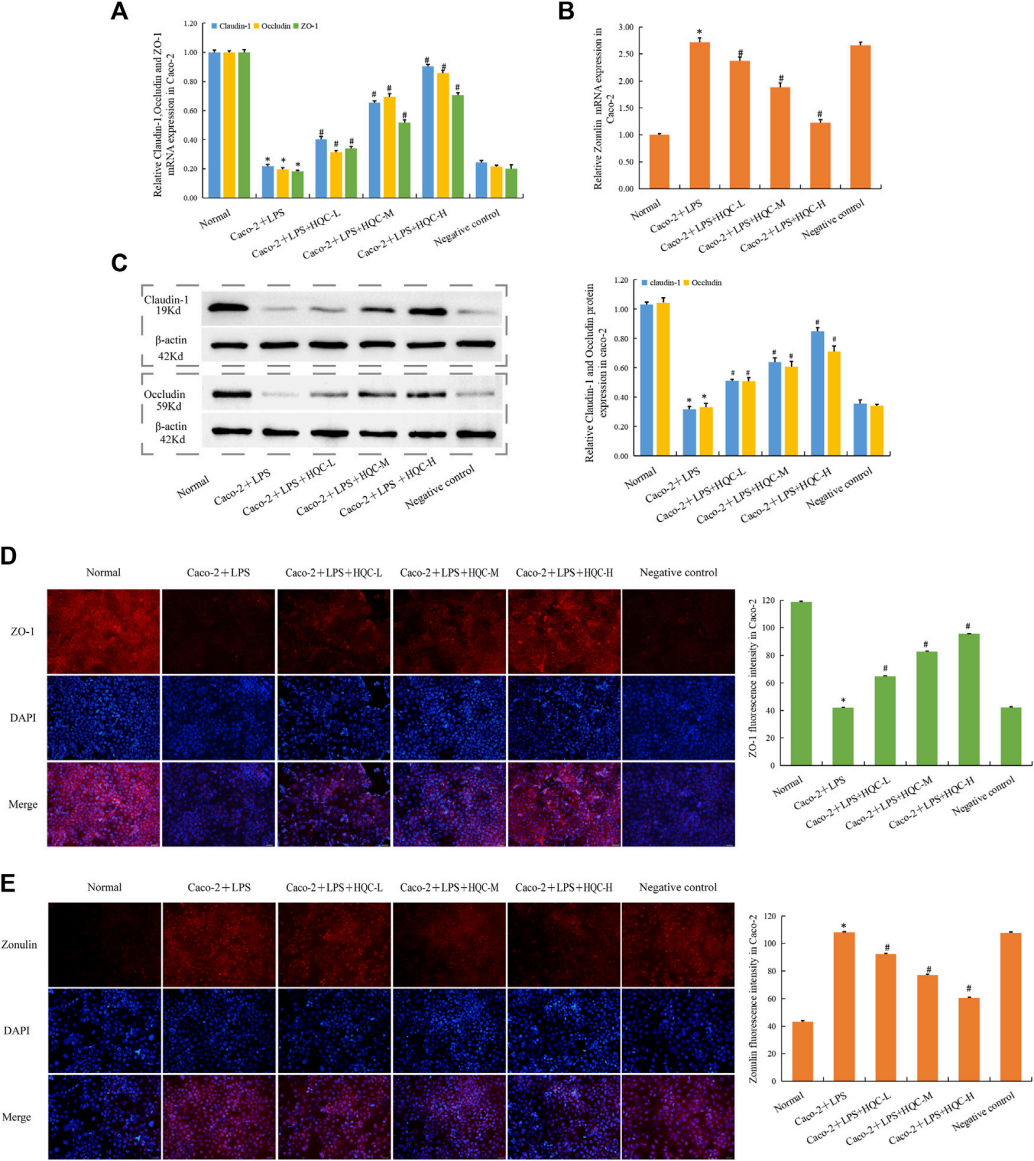
HQC treatment restored intestinal barrier damage in Caco-2 cells **(A)**, The mRNA expression of ZO-1, Occludin, and Claudin-1 in Caco-2 cells; **(B)**, The mRNA expression of Zonulin in Caco-2 cells; **(C)**, The protein expression of Occludin, and Claudin-1 in Caco-2 cells; **(D)**, Immunofluorescence detection ZO-1 expression in Caco-2 cells (scale bar = 50 μm); **(E)**, Immunofluorescence detection Zonulin expression in Caco-2 cells (scale bar = 50 μm). *p < 0.05 vs. Normal, #*p* < 0.05 vs. Caco-2+LPS; for **(A–C)**, *n* = 5; for **(D and E)**, *n* = 3.

In Caco-2 cells, compared with the normal group, the mRNA expression of ZO-1, Occludin and Claudin-1 in Caco-2+LPS group decreased (Figure 6A), while the mRNA expression of Zonulin increased (Figure 6B), and HQC treatment reversed the above indicators. WB results showed that HQC upregulated the protein expression of decreased Occludin and Claudin-1 in LPS-treated Caco-2 cells (Figure 6C). Immunofluorescence results showed that HQC upregulated the level of ZO-1 (Figure 6D) and downregulated the level of Zonulin (Figure 6E) in LPS-treated Caco-2. Compared with the normal group, intestinal barrier injury was observed in Caco-2+LPS group, and HQC treatment could alleviate the dysfunction in the intestinal epithelial barrier caused by inflammation in Caco-2 cells treated with LPS.

### 3.7 LPS aggravated RA pathology and HQC inhibits LPS generation

**FIGURE 7:**
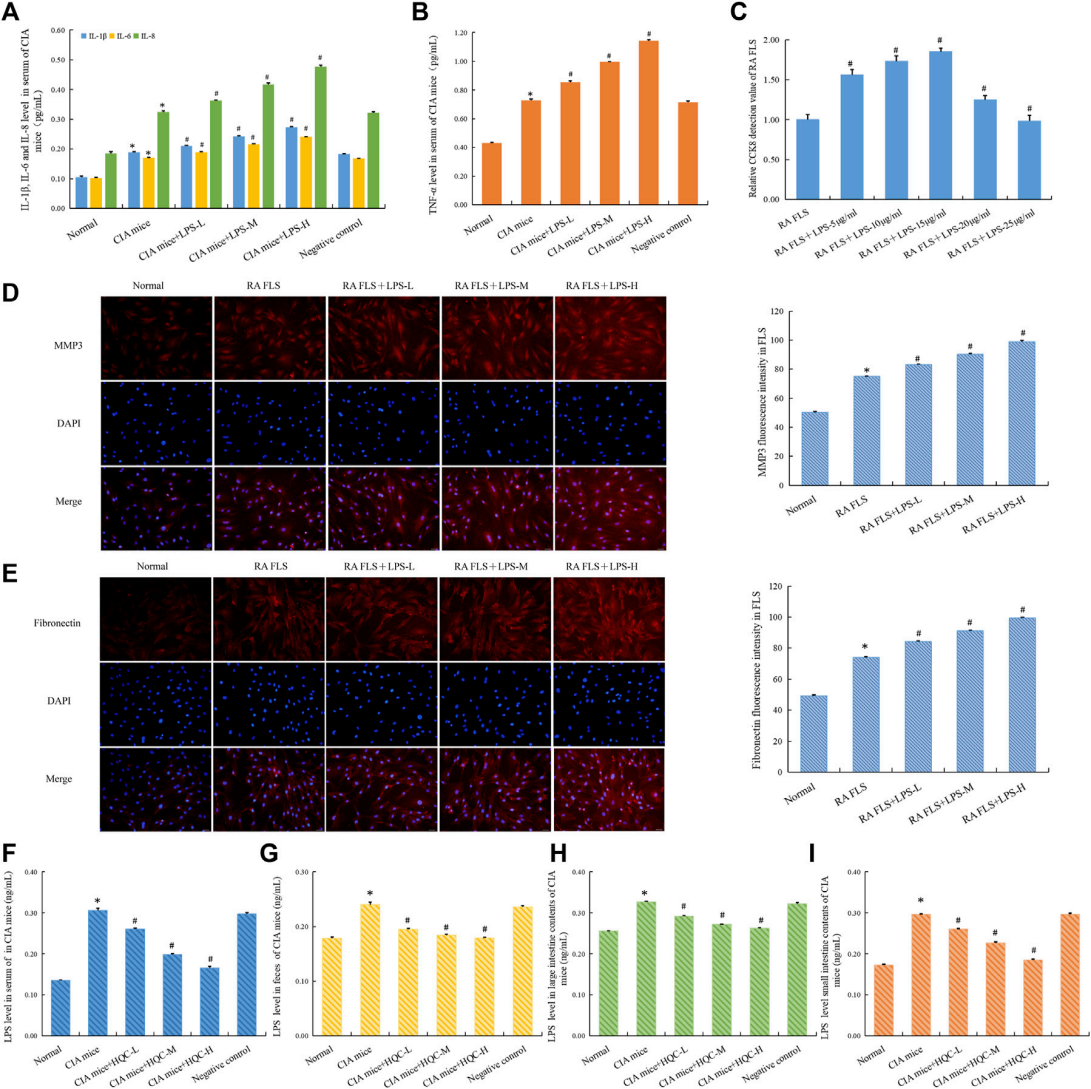
LPS aggravated RA pathology and HQC inhibits LPS generation **(A)**, IL-1β, IL-6, and IL-8 level in the serum; **(B)**, TNF-α level in the serum; **(C)**, CCK-8 detection of the effect of LPS on the proliferation of RA-FLS; **(D)**, Immunofluorescence detection MMP3 expression in FLS; **(E)**, Immunofluorescence detection Fibronectin expression in FLS; **(F)**, LPS level in serum; **(G)**, LPS level in feces; **(H)**, LPS level in the large intestinal contents; **(I)**, LPS level in the small intestinal contents *p < 0.05 vs. Normal, #*p* < 0.05 vs. CIA mice, *n* = 3.

The serum levels of inflammatory factors IL-1β, IL-6, IL-8 and TNF-α in mice in each group were detected by ELISA, and the results showed that LPS could increase the levels of these inflammatory factors to varying degrees (Figures 7A,B). At the cellular level, CCK8 results showed that LPS at different doses promoted the proliferation of RA FLS (Figure 7C). Immunofluorescence results showed that compared with the model group, the expressions of MMP3 and Fibronectin were increased in the CIA + LPS group (Figures 7D,E). ELISA was used to detect LPS level in serum (Figure 7F), feces (Figure 7G), small intestine contents (Figure 7H) and large intestine contents (Figure 7I). Compared with the normal group, the CIA model group showed an increase in LPS level, while HQC downregulated LPS level. These results suggest that HQC has a regulatory effect on LPS, and HQC may alleviate RA by regulating LPS.

### 3.8 HQC exerted anti RA effects by regulating intestinal flora and inhibiting LPS entry into peripheral blood

**FIGURE 8:**
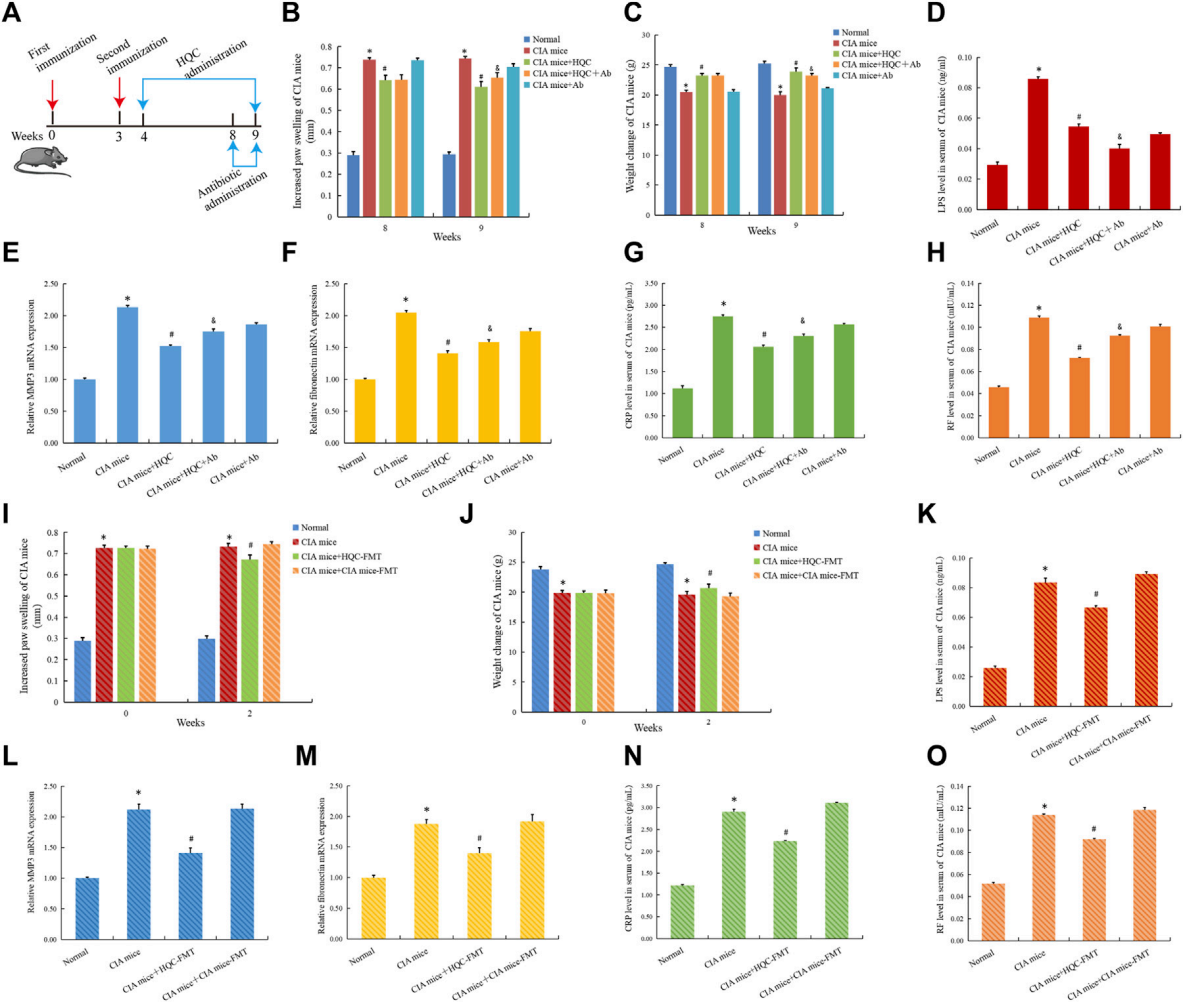
HQC exerted anti RA effects by regulating intestinal flora and inhibiting LPS entry into peripheral blood **(A)**, The chart of animal treatments; **(B)**, Foot swelling from week eight to week 9; **(C)**, Body weight in mice from week eight to week 9; **(D)**, LPS levels in serum; **(E)**, The mRNA expression of MMP3 in the synovial tissue; **(F)**, The mRNA expression of Fibronectin in the synovial tissue; **(G)**, CRP level in serum; **(H)**, RF level in serum; **(I)**, Foot swelling from week 0 to week 2; **(J)**, Body weight in mice from week 0 to week 2; **(K)**, LPS level in serum; **(L)**, The mRNA expression of MMP3 in the synovial tissue; **(M)**, The mRNA expression of Fibronectin in the synovial tissue; **(N)**, CRP level in serum; **(O)**, RF level in serum. *p < 0.05 vs. Normal, #*p* < 0.05 vs. CIA mice, &*p* < 0.05 vs. CIA mice + HQC; for **(B,C,I,J)**, *n* = 10; for **(D,G,H,K,N,O)**, *n* = 3; for **(E,F,L,M)**, *n* = 5.

To further confirm the role of intestinal flora in improving RA pathology of HQC, We used anti-biodepletion intestinal flora and HQC-FMT methods to investigate whether HQC suppresses RA pathology through intestinal flora. Antibiotic (neomycin sulfate (200 mg/kg), ampicillin (200 mg/kg), vancomycin (100 mg/kg) and metronidazole (200 mg/kg) was administered for 1 week (Figure 8A). Compared with the normal group, foot swelling increased (Figure 8B) and body weight decreased (Figure 8C) in the CIA model group, and HQC reversed the above indexes, but antibiotic depletion of intestinal flora could weaken the effects of HQC on the above indexes. HQC can reduce LPS level, and LPS level decrease after antibiotic depletion of intestinal flora (Figure 8D). Compared with the normal group, the expression of MMP3 (Figure 8E) and Fibronectin (Figure 8F) in the synovial tissue and the levels of CRP (Figure 8G) and RF (Figure 8H) in serum of CIA mice were increased, and HQC reversed the above indexes, but the depletion of intestinal flora by antibiotic could reduce the influence of HQC on the above indexes in the CIA model mice. These results indicated that HQC exerted anti-RA effect through intestinal flora.

In the HQC-FMT experiment, compared with the normal group, foot swelling increased (Figure 8I) and body weight decreased (Figure 8J) in the CIA model group, and HQC-FMT reversed the above indexes. HQC-FMT can reduce LPS levels (Figure 8K). Compared with the normal group, the expressions of MMP3 (Figure 8L) and Fibronectin (Figure 8M) in the synovial tissue and the CRP (Figure 8N) and RF (Figure 8O) levels in serum of CIA mice were increased, and HQC-FMT reversed the above indexes, The above results further indicated that HQC played an anti-RA role by regulating intestinal flora.

## 4 Discussion

RA is a common autoimmune disease, and anti rheumatic drugs have made great progress in treating RA. However, we still need to deeply understand the pathogenesis of RA and achieve targeted treatment of RA. More evidence shows that the intestinal flora undergoes changes before RA occurs, indicating a close relationship between intestinal flora and the occurrence of RA (Vaahtovuo et al., 2008). Intestinal microbes and their metabolites affect human health through intestinal epithelial immunity (Levy et al., 2017). Dysfunction of intestinal flora leads to immune inflammatory response and changes in intestinal barrier function, thereby promoting the occurrence of RA (Maeda et al., 2016; Wang H. et al., 2021). However, the exact mechanism by which intestinal flora affects RA remains unclear.

In RA model mice, intestinal barrier function is impaired before the onset of arthritis and is associated with the occurrence of RA (Akdis, 2021). Intestinal epithelial tight junction proteins Occludin, ZO-1, Claudin-1 and regulatory factor Zonulin are the main markers to evaluate whether the intestinal barrier is damaged. Specifically targeting Zonulin therapy prevents the transfer of immune cells from the gut to the joints, partially preventing the development of arthritis. This suggests that regulating intestinal barrier function may help prevent or treat RA (Tajik et al., 2020). However, the molecular mechanism of regulating the intestinal barrier to improve RA is still unclear, and it is an emerging RA research field.

TCM has become a hot research topic in RA treatment because of its multiple active ingredients. HQC has obvious anti-inflammatory effect, but the specific mechanism needs to be clarified. CIA mice have pathologic characteristics similar to human RA, and they have been widely used in the testing of RA therapeutic drugs and RA pathological studies (Choudhary et al., 2018). Therefore, this study established a CIA mouse model to explore whether HQC can improve RA by regulating intestinal flora and intestinal barrier, thereby inhibiting LPS entry into the blood.

In this experiment, HQC significantly affected CIA mice. After HQC treatment, the swelling degree, histopathological injury degree and inflammation degree of CIA mice’s hind limbs were significantly reduced. Additionally, data on arthritis scores, body weight, and incidence corroborated this. We conducted 16SrRNA sequencing on the large and small intestine contents samples of different groups of mice, our findings indicated an insignificant variance in α diversity and significant difference in β diversity between the normal group, CIA model group and HQC treatment group. At the genus level, there were significant changes in the microbiota of the CIA model group compared to the normal group, The relative abundance of Gram-negative bacteria *Muribaculaceae*, *Helicobacter*, *Prevotellaceae_UCG-001*, *Muribaculum*, *Rikenella* and *Desulfovibrio* has increased significantly. The relative abundance of Gram-positive bacteria *Lactococcus*, *Candidatus_Arthromitus*, *Clostridia_UCG-014*, *Bifidobacterium* and *Lachnoclostridium* has decreased significantly. The HQC treatment group could partially restore the changes in the microbiota of the CIA model group. At the family level, there were significant changes in the microbiota of the CIA model group compared to the normal group, The relative abundance of Gram-negative bacteria *Muribaculaceae*, *Helicobacteraceae*, *Rikenellaceae*, *Oscillospiraceae*, *Prevotellaceae* and *Desulfovibrionaceae* has increased significantly*.* The relative abundance of Gram-positive bacteria *Ruminococcaceae, Erysipelotrichaceae, Clostridiaceae, Eggerthellaceae, Clostridia_UCG-014, Bifidobacteriaceae* and *Sutterellaceae* has decreased significantly. Among them, excessive proliferation of *Prevotellaceae* may affect the function of immune cells in mucosal and systemic sites, resulting in the occurrence of RA. *Helicobacter* produces urease, which stimulates B cells and leads to the production of rheumatoid factors (Yamanishi et al., 2006).

PICRUST software predicted the function of large intestine contents and small intestine contents, and found that after HQC intervention, the LPS production pathway in mice was significantly weakened, and the short-chain fatty acid and aromatic fatty acid production pathway was significantly enhanced. Short chain fatty acids (SCFAs) are the metabolites of the gut flora and have various physiological effects on the host. SCFAs can regulate the differentiation of B and T cells, thereby reducing the production of RA autoantibodies (Rosser et al., 2020; Takahashi et al., 2020). SCFAs can also enhance intestinal barrier function and promote intestinal homeostasis (Lewis et al., 2010). Tryptophan in aromatic fatty acids inhibits pro-inflammatory responses in macrophages and enhances intestinal barrier function (Bansal et al., 2010). We suspected that HQC may play a role by regulating LPS and metabolites of the intestinal flora. Combined with the results of large intestine, small intestine tissue and Caco-2 cells, the expression of Claudin-1, ZO-1 and Occludin in CIA model group decreased, and the expression of Zonulin increased compared with the normal group, indicating that intestinal barrier damage in CIA model group was recovered by HQC treatment.

When the intestinal flora is disturbed, especially when Gram-negative bacteria over reproduce, large amounts of LPS are produced. Dysfunction of intestinal flora may lead to changes in intestinal barrier function. Pro-inflammatory microbial products, such as LPS, then cross the damaged barrier with cytokines and enter the circulation, causing inflammation throughout the body. Studies have shown that elevated LPS can be recognized by TLR4 and then stimulate the NF-κB signaling pathway, causing inflammation (Zhao et al., 2021). LPS can activate complement alternative pathway, this pathway is critical in the development of autoimmune arthritis, LPS may promote the occurrence of arthritis through this pathway (Murayama et al., 2015). We found LPS level increased in the CIA model group, and LPS level decreased in the HQC treatment group, indicating that HQC has a regulatory effect on LPS. LPS increased the expression of RA pathologic factors and inflammatory factors, and FLS proliferation. Findings indicated that LPS was a factor in the development of RA. In summary, HQC alleviates RA by modulating LPS.

To investigate whether intestinal bacteria mediate the antiarthritic effect of HQC, we set up a pseudo-sterile CIA mice experimental group. The results indicated that when the intestinal flora of the HQC treatment group was disturbed by antibiotic, the effect of HQC on alleviating arthritis was reduced. These results suggested that the antiarthritic effect of HQC depended on the regulation of intestinal flora. In order to further prove this view, we conducted an HQC-FMT experiment, and the results showed that HQC-FMT could reduce the levels of LPS, RF and CRP in the CIA model group. It is suggested that HQC has an anti-RA function through the regulation of intestinal flora.

Our investigation delved into how HQC functions in RA treatment, focusing on the “gut-peripheral blood-joint” axis. We explored the mechanism of HQC in RA treatment from the perspective of “gut-peripheral blood-joint” axis, which will offer new insights into treating RA through the regulation of intestinal flora and intestinal barrier. LPS also has an impact on RA pathology. However, the specific mechanism of LPS promoting RA is still unclear, and further in-depth research is needed.

## Data Availability

The original contributions presented in the study are included in the article/Supplementary Material, further inquiries can be directed to the corresponding authors.
