# Supplementary material for: Huangqin Qingre Chubi Capsule inhibits rheumatoid arthritis by regulating intestinal flora and improving intestinal barrier
Source: Front Pharmacol. 2024 Jun 26;15:1422245. doi: 10.3389/fphar.2024.1422245 (PMC11233690; doi:10.3389/fphar.2024.1422245)

Claudin (abcam: ab307692) 19KD; β-actin (abcam: ab8226) 42KD

Sample: Caco2 Cell

Repeat 1

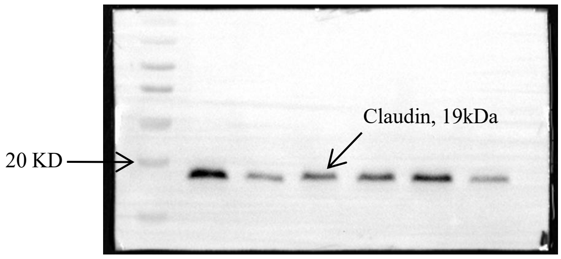

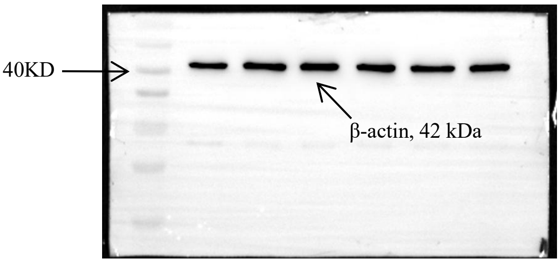

Repeat 2

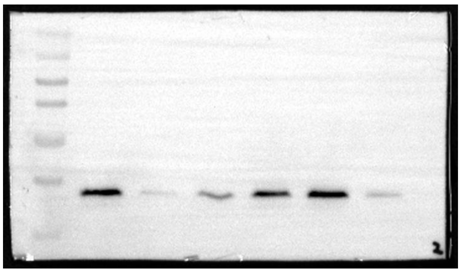

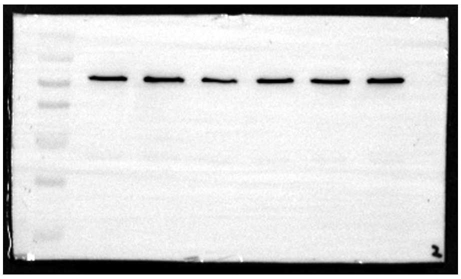

Repeat 3

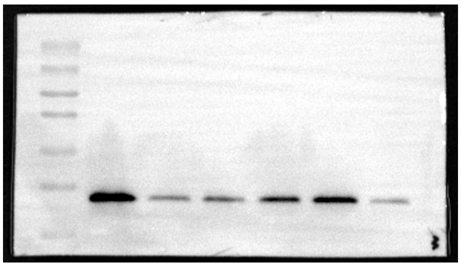

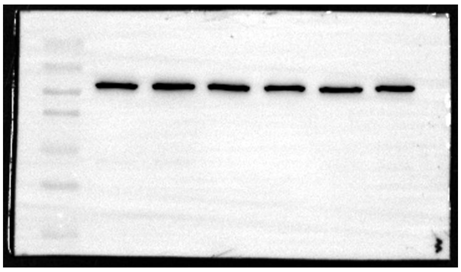

Repeat 4

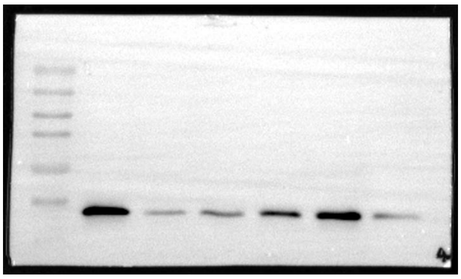

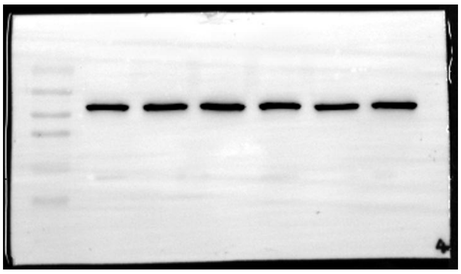

Repeat 5

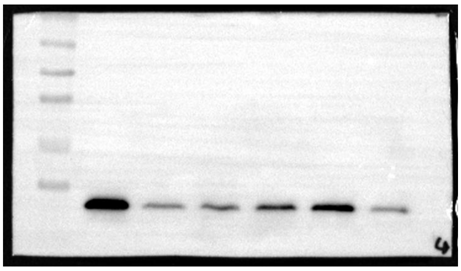

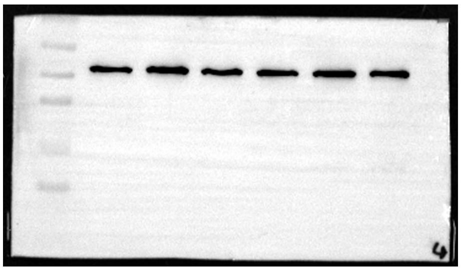

Sample: Large intestine

Repeat 1

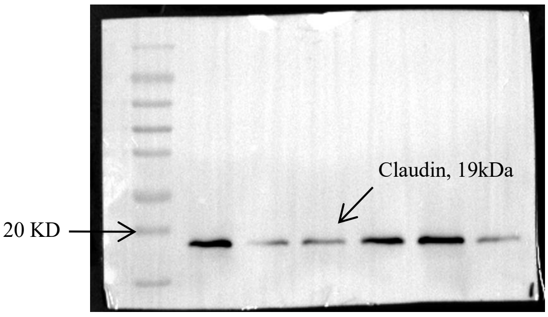

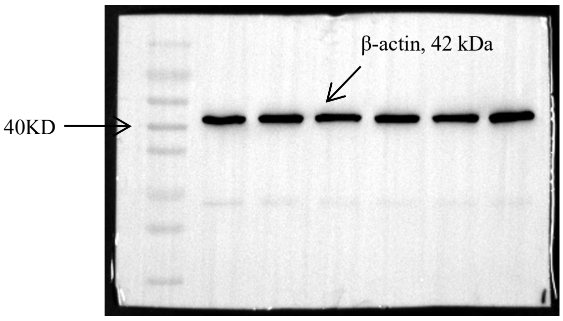

Repeat 2

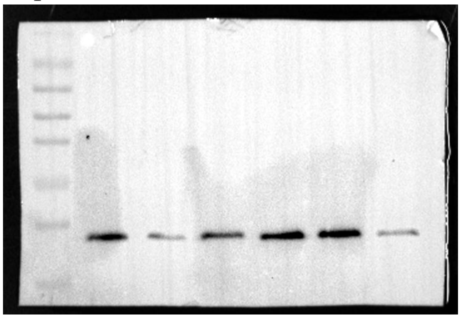

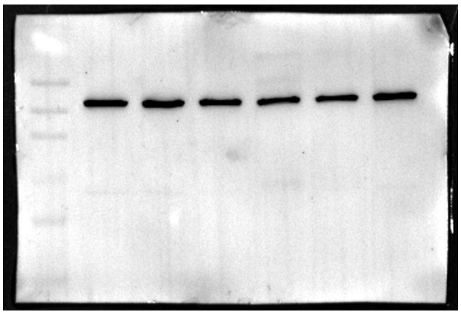

Repeat 3

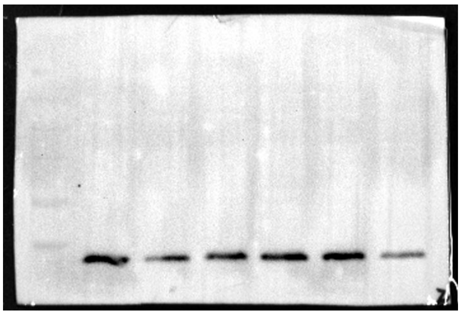

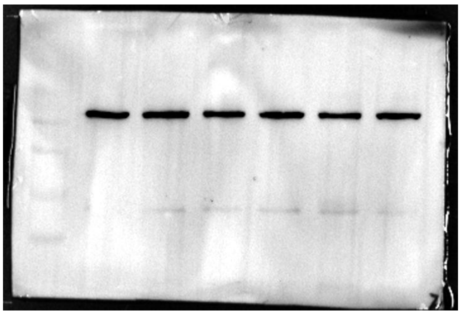

Repeat 4

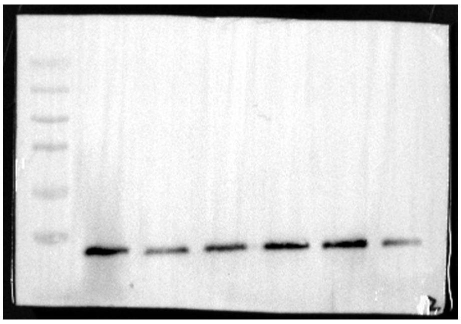

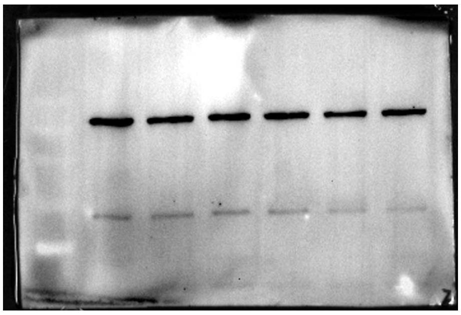

Repeat 5

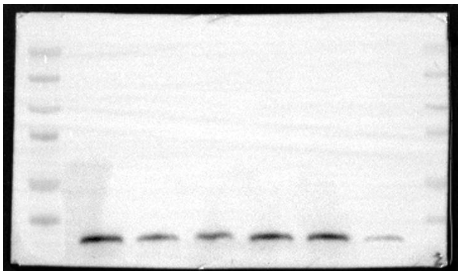

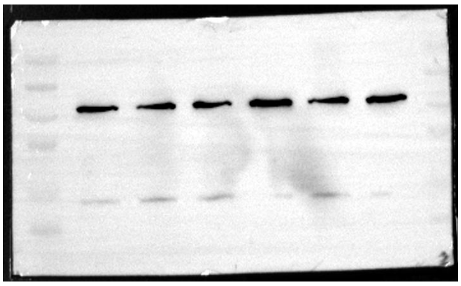

Sample: Small intestine

Repeat 1

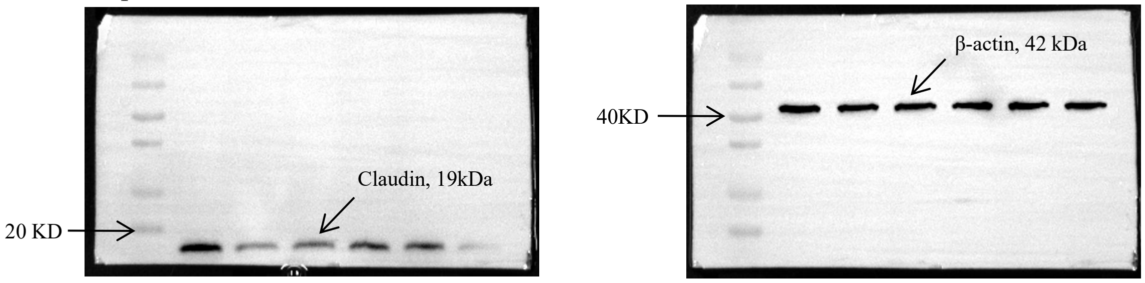

Repeat 2

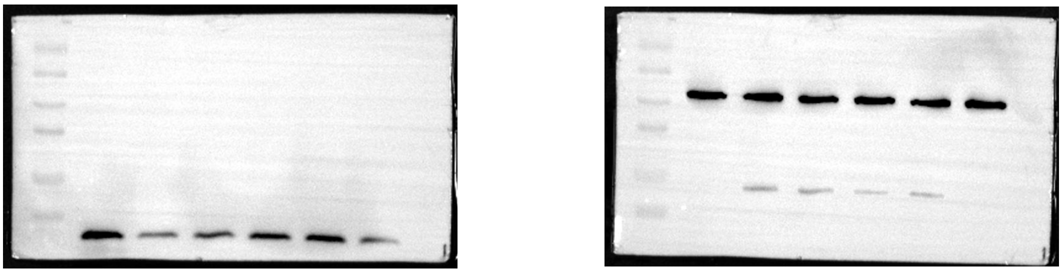

Repeat 3

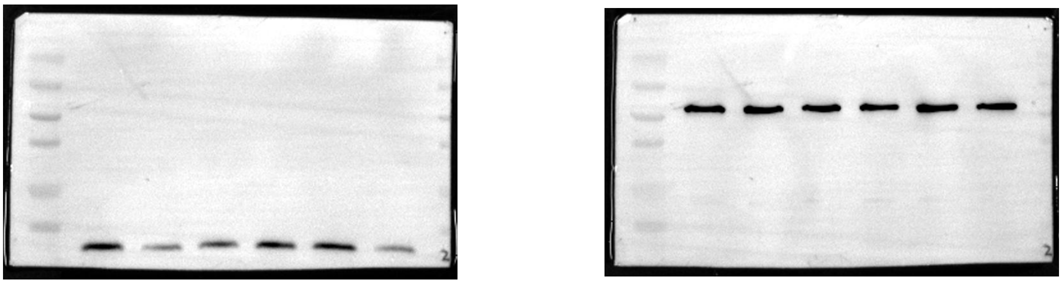

Repeat 4

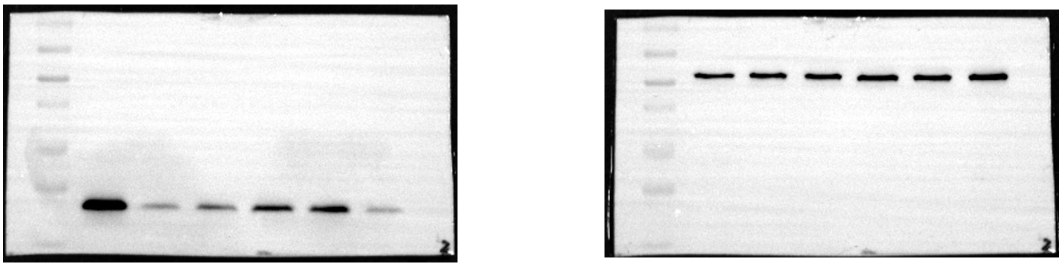

Repeat 5

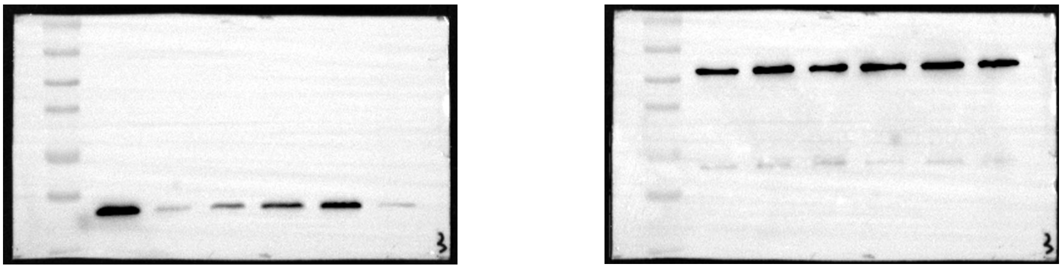

Occludin (abcam: ab215327) 59KD; β-actin (abcam: ab8226) 42KD

Sample: Caco2 Cell

Repeat 1

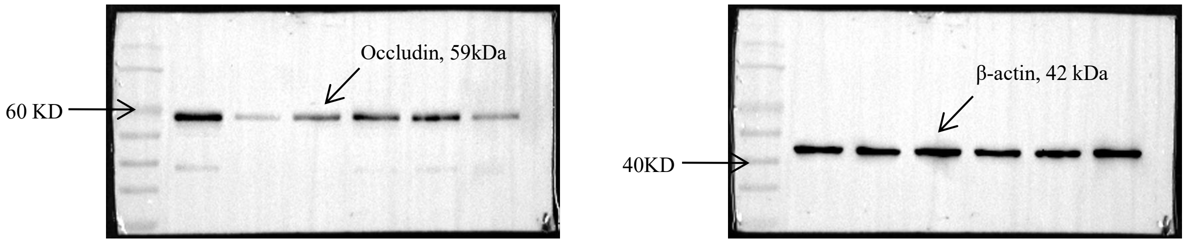

Repeat 2

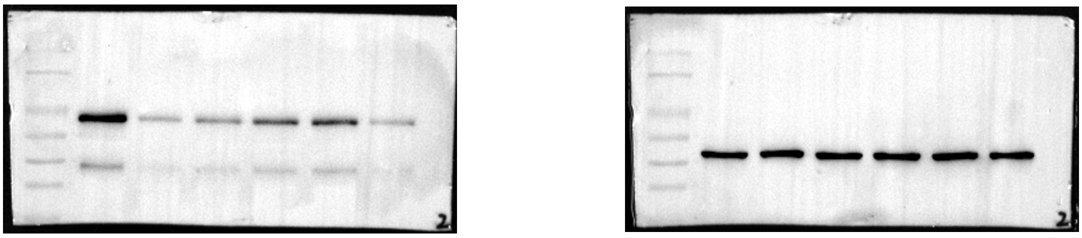

Repeat 3

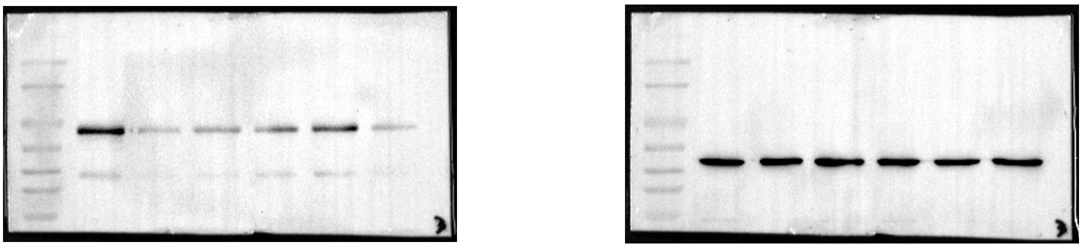

Repeat 4

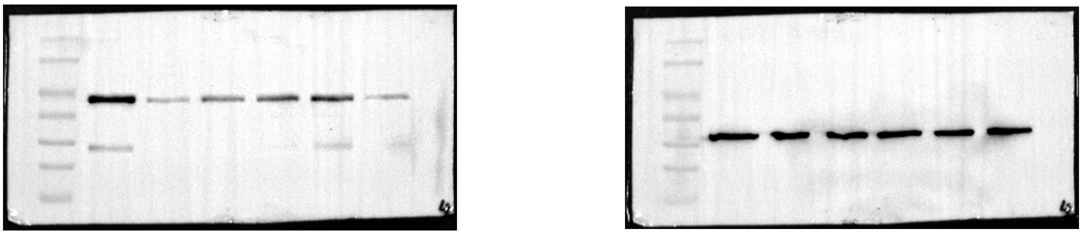

Repeat 5

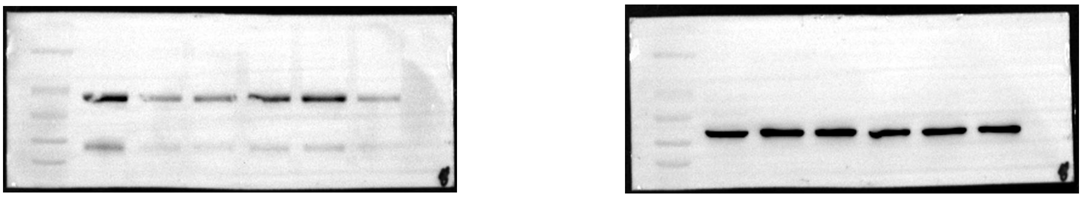

Sample: Large intestine

Repeat 1

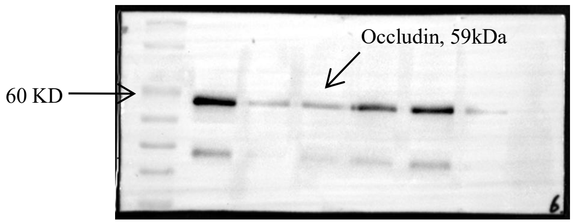

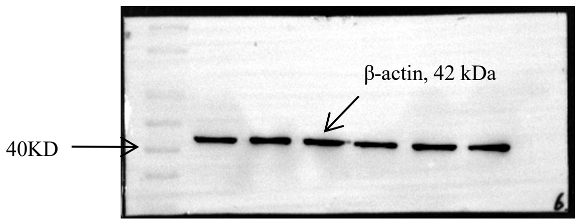

Repeat 2

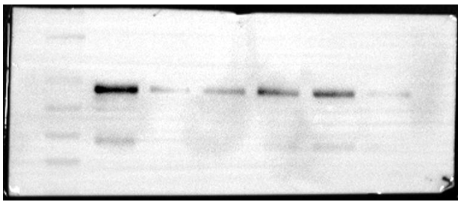

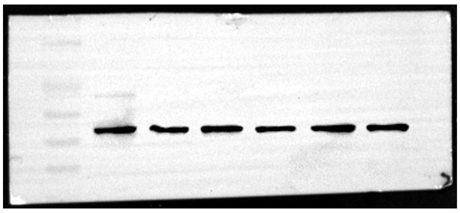

Repeat 3

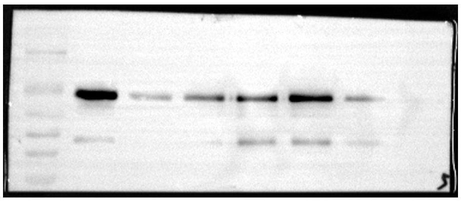

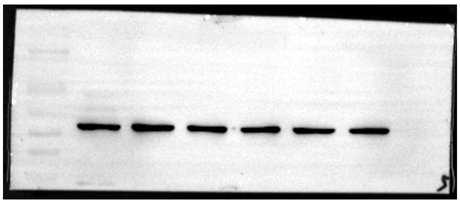

Repeat 4

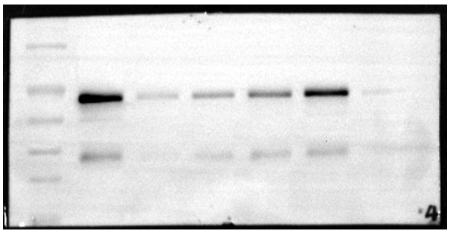

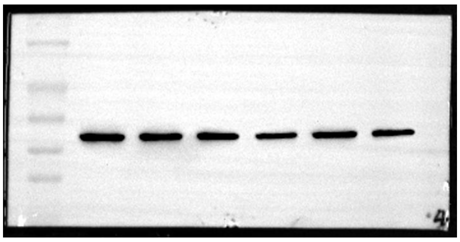

Repeat 5

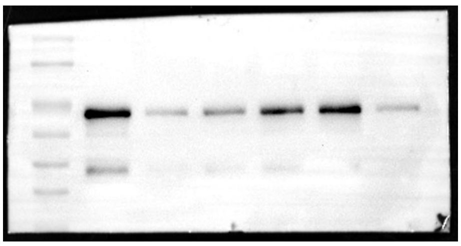

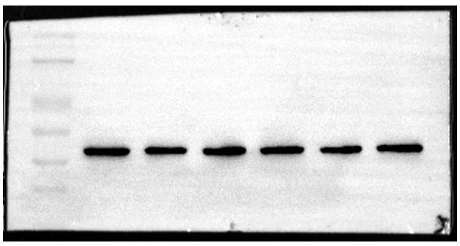

Sample: Small intestine

Repeat 1

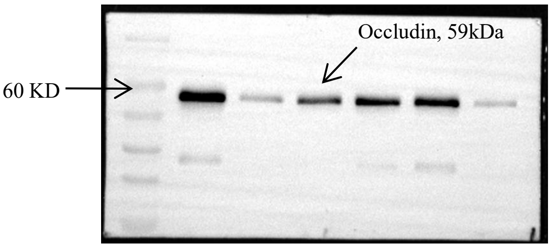

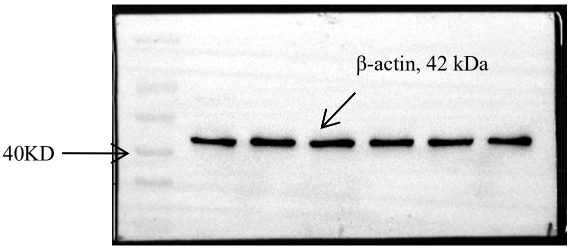

Repeat 2

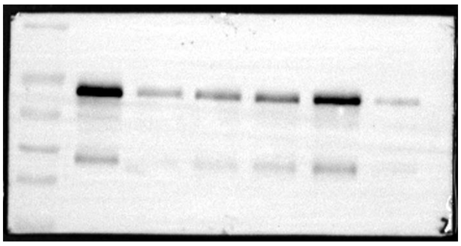

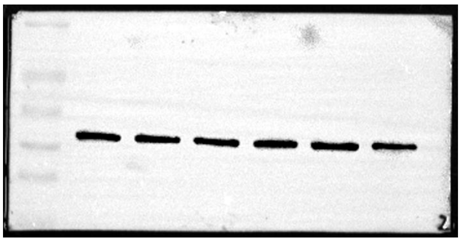

Repeat 3

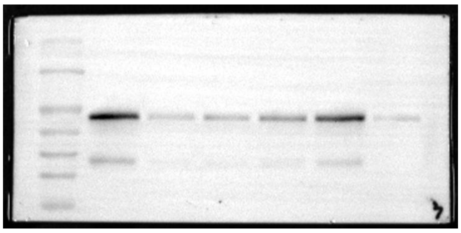

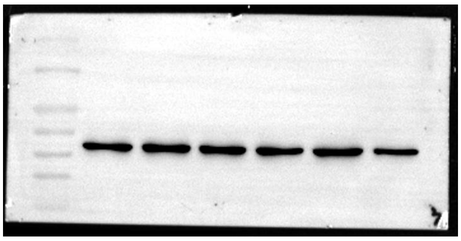

Repeat 4

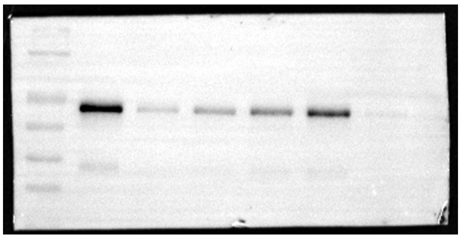

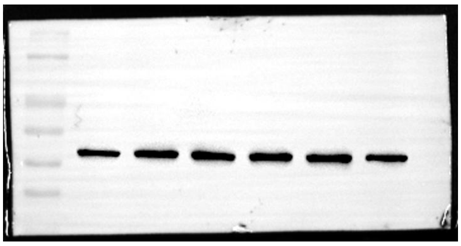

Repeat 5

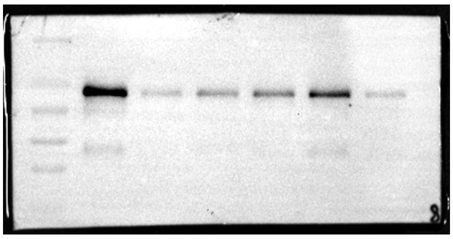

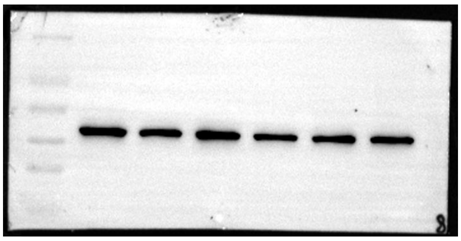

Supplement: Supplementary file 2 [file DataSheet9.PDF]
